# Impact of Nutrition on Female Reproductive Disorders

**DOI:** 10.3390/nu15214576

**Published:** 2023-10-27

**Authors:** Pasquapina Ciarmela, Stefania Greco

**Affiliations:** Department of Experimental and Clinical Medicine, Università Politecnica delle Marche, 60126 Ancona, Italy

The female reproductive system is a delicate and complex system in the body that can be affected by many disorders. Many pathologies may occur during the female reproductive age, from menarche through menopause, and may impair women’s fertility and pregnancy outcomes [1].

Nutrition may benefit different female organs, providing an important protective effect on the reproductive system, while specific nutritional deprivation may result in gynecological and fertility health problems [2,3,4].

This Special Issue of *Nutrients* collected 11 papers (6 original articles, 2 reviews, 1 opinion, 1 systemic review and 1 commentary) focusing on the impact of nutrition on female reproductive disorders.

Among nutrients, vitamins are the most known elements essential for human health. Our body needs only tiny amounts of vitamins; however, they have a big role in maintaining a healthy status, while the deficiencies of different vitamins can harm health in different ways [5,6,7].

The present Special Issue covers three articles on vitamin D [8,9,10] and one on vitamin A [8]. The articles on vitamin D are two original articles [9,10] and one commentary [11]. The original articles report two different populations, Swedes [9] and Finns [10], regarding the effect of vitamin D insufficiency on female infertility. These findings are in line with recent literature, as reported in the commentary by Grzeczka et al., which provides an update on the importance of vitamin D and vitamin D deficiency in the oocyte and the follicular microenvironment, wherein vitamin D deficiency has been associated with decreased live birth rates among women undergoing in vitro fertilization [11].

In an in vivo study, Li and colleagues demonstrated that dysbacteriosis of the intestinal flora due to colitis may reduce ovarian function in mice due to reduced vitamin A absorption [8].

The intestinal flora has also been studied in another study included in this Special Issue that reports its important role in pregnancy outcomes for embryo quality as a consequence of short-chain fatty acids produced by the gut microbiota [12].

Two articles focused on carbohydrates [13,14]. Salcedo and colleagues wrote an opinion article that encourages a lifestyle-based intervention such as therapeutic carbohydrate restriction as prevention and potential treatment of all gynecologic disorders that lead to Abnormal Uterine Bleeding [13]. On the other hand, the disaccharide trehalose is a naturally occurring carbohydrate that may be useful to promote good health because of its properties that do not stimulate rapid increases in blood glucose. The experimental study of Kang et al. showed that trehalose suppresses lysosomal anomalies in supporting cells of oocytes, suggesting a possible therapeutic effect on female fertility, although the clinical use remains speculative and limited [14].

Polycystic Ovary Syndrome (PCOS) has been considered in two papers [15,16]. A deficiency in dietary fiber can be associated with a wide range of metabolic abnormalities and with an altered gut microbial ecosystem that can be associated with reproductive abnormalities, including PCOS. Therefore, Leung and colleagues wrote a systematic review that reported a meta-analysis of available evidence on the dietary fiber intake level in PCOS patients [15].

In the second paper about PCOS, Guarano and colleagues reviewed the existing literature to report the efficacy and the limitations of alpha lipoic acid in PCOS treatment since alpha lipoic acid is considered a promising therapeutic strategy for this very complex syndrome due to its multiple actions, including anti-inflammatory, antioxidant, and insulin-sensitizing [16].

With regard to uterine disease, the review of Li et al. supports the potential of melatonin supplements in the development of endometriosis, although more clinical trials are needed to confirm its therapeutic effects and safety [17]. The last paper is an in vitro study that explored the potential of a specific fruit extract on a malignant gynecological disease. It has been observed that the extract of the cultivar Romina strawberry, characterized by a high content of anthocyanins and antioxidant capacity, can reduce the spheroid formation and the extracellular matrix apposition of leiomyoma cell line cultured in three-dimensional agarose gel [18].

Overall, the knowledge of the potential of nutrients in promoting human health and wellbeing is rapidly improving. We are glad that this collection includes interesting studies on the benefits of nutrition in the prevention and therapy of female reproductive disorders.

## References

[1] Deffieux X., Rousset-Jablonski C., Gantois A., Brillac T., Maruani J., Maitrot-Mantelet L., Mignot S., Gaucher L., Athiel Y., Baffet H. (2023). Examen pelvien en gynécologie et obstétrique: Recommandations pour la pratique clinique [Pelvic exam in gynecology and obstetrics: Guidelines for clinical practice]. Gynecol. Obstet. Fertil. Senol..

[2] Izetbegovic S., Alajbegovic J., Mutevelic A., Pasagic A., Masic I. (2013). Prevention of diseases in gynecology. Int. J. Prev. Med..

[3] Kussmann M., Fay L.B. (2008). Nutrigenomics and personalized nutrition: Science and concept. Per. Med..

[4] Tan B.L., Norhaizan M.E., Liew W.P. (2018). Nutrients and Oxidative Stress: Friend or Foe?. Oxid. Med. Cell Longev..

[5] Elhusseini H., Lizneva D., Gavrilova-Jordan L., Eziba N., Abdelaziz M., Brakta S., Halder S., Al-Hebdy A., Gower S. (2017). Vitamin d and female reproduction. A Critical Evaluation of Vitamin D: Basic Overview.

[6] Shenkin A. (2006). Micronutrients in health and disease. Postgrad. Med. J..

[7] Ryan-Harshman M., Aldoori W. (2005). Health benefits of selected vitamins. Can Fam. Physician..

[8] Li Z., Chen C., Yu W., Xu L., Jia H., Wang C., Pei N., Liu Z., Luo D., Wang J. (2023). Colitis-Mediated Dysbiosis of the Intestinal Flora and Impaired Vitamin A Absorption Reduce Ovarian Function in Mice. Nutrients.

[9] Maaherra Armstrong P., Augustin H., Bärebring L., Osmancevic A., Bullarbo M., Thurin-Kjellberg A., Tsiartas P. (2023). Prevalence of Vitamin D Insufficiency and Its Determinants among Women Undergoing In Vitro Fertilization Treatment for Infertility in Sweden. Nutrients.

[10] Lumme J., Morin-Papunen L., Pesonen P., Sebert S., Hyppönen E., Järvelin M.-R., Herzig K.-H., Ojaniemi M., Niinimäki M. (2023). Vitamin D Status in Women with a History of Infertility and Decreased Fecundability: A Population-Based Study. Nutrients.

[11] Grzeczka A., Graczyk S., Skowronska A., Skowronski M.T., Kordowitzki P. (2022). Relevance of Vitamin D and Its Deficiency for the Ovarian Follicle and the Oocyte: An Update. Nutrients.

[12] Yao X., Dong S., Guan W., Fu L., Li G., Wang Z., Jiao J., Wang X. (2023). Gut Microbiota-Derived Short Chain Fatty Acids Are Associated with Clinical Pregnancy Outcome in Women Undergoing IVF/ICSI-ET: A Retrospective Study. Nutrients.

[13] Salcedo A.C., Yun J., Carter C., Hart E. (2023). Therapeutic Carbohydrate Restriction as a Metabolic Modality for the Prevention and Treatment of Abnormal Uterine Bleeding. Nutrients.

[14] Kang W., Ishida E., Amita M., Tatsumi K., Yonezawa H., Yohtsu M., Katano D., Onozawa K., Kaneko E., Iwasaki W. (2022). Trehalose Suppresses Lysosomal Anomalies in Supporting Cells of Oocytes and Maintains Female Fertility. Nutrients.

[15] Leung W.T., Tang Z., Feng Y., Guan H., Huang Z., Zhang W. (2022). Lower Fiber Consumption in Women with Polycystic Ovary Syndrome: A Meta-Analysis of Observational Studies. Nutrients.

[16] Guarano A., Capozzi A., Cristodoro M., Di Simone N., Lello S. (2023). Alpha Lipoic Acid Efficacy in PCOS Treatment: What Is the Truth?. Nutrients.

[17] Li Y., Hung S.-W., Zhang R., Man G.C.-W., Zhang T., Chung J.P.-W., Fang L., Wang C.-C. (2022). Melatonin in Endometriosis: Mechanistic Understanding and Clinical Insight. Nutrients.

[18] Greco S., Pellegrino P., Giampieri F., Capocasa F., Delli Carpini G., Battino M., Mezzetti B., Giannubilo S.R., Ciavattini A., Ciarmela P. (2023). The In Vitro Effects of Romina Strawberry Extract on 3D Uterine Leiomyosarcoma Cells. Nutrients.

